# Additional Value of PET Radiomic Features for the Initial Staging of Prostate Cancer: A Systematic Review from the Literature

**DOI:** 10.3390/cancers13236026

**Published:** 2021-11-30

**Authors:** Priscilla Guglielmo, Francesca Marturano, Andrea Bettinelli, Michele Gregianin, Marta Paiusco, Laura Evangelista

**Affiliations:** 1 Nuclear Medicine Unit, Veneto Institute of Oncology IOV—IRCCS, 31033 Castelfranco Veneto, Italy; priscilla.guglielmo@iov.veneto.it (P.G.); michele.gregianin@iov.veneto.it (M.G.); 2 Medical Physics Unit, Veneto Institute of Oncology IOV—IRCCS, 32168 Padova, Italy; francesca.marturano@iov.veneto.it (F.M.); andrea.bettinelli@iov.veneto.it (A.B.); marta.paiusco@iov.veneto.it (M.P.); 3 Nuclear Medicine Unit, Department of Medicine DIMED, University of Padova, 32168 Padova, Italy

**Keywords:** prostate cancer, PET, radiomics, staging

## Abstract

**Simple Summary:**

Prostate cancer (PCa) is one of the most frequent malignancies diagnosed in men and its prognosis depends on the stage at diagnosis. Molecular imaging, namely PET/CT or PET/MRI using prostate-specific radiotracers, has gained increasing application in accurately evaluating PCa at staging, especially in cases of high-risk disease, and it is now also recommended by international guidelines. Radiomic analysis is an emerging research field with a high potential to offer non-invasive and longitudinal biomarkers for personalized medicine, and several applications have been described in oncology patients. In this review, we discuss the available evidence on the role of radiomic analysis in PCa imaging at staging, exploring two different hybrid imaging modalities, such as PET/CT and PET/MRI, and the whole spectrum of radiotracers involved.

**Abstract:**

We performed a systematic review of the literature to provide an overview of the application of PET radiomics for the prediction of the initial staging of prostate cancer (PCa), and to discuss the additional value of radiomic features over clinical data. The most relevant databases and web sources were interrogated by using the query “prostate AND radiomic* AND PET”. English-language original articles published before July 2021 were considered. A total of 28 studies were screened for eligibility and 6 of them met the inclusion criteria and were, therefore, included for further analysis. All studies were based on human patients. The average number of patients included in the studies was 72 (range 52–101), and the average number of high-order features calculated per study was 167 (range 50–480). The radiotracers used were [68Ga]Ga-PSMA-11 (in four out of six studies), [18F]DCFPyL (one out of six studies), and [11C]Choline (one out of six studies). Considering the imaging modality, three out of six studies used a PET/CT scanner and the other half a PET/MRI tomograph. Heterogeneous results were reported regarding radiomic methods (e.g., segmentation modality) and considered features. The studies reported several predictive markers including first-, second-, and high-order features, such as “kurtosis”, “grey-level uniformity”, and “HLL wavelet mean”, respectively, as well as PET-based metabolic parameters. The strengths and weaknesses of PET radiomics in this setting of disease will be largely discussed and a critical analysis of the available data will be reported. In our review, radiomic analysis proved to add useful information for lesion detection and the prediction of tumor grading of prostatic lesions, even when they were missed at visual qualitative assessment due to their small size; furthermore, PET radiomics could play a synergistic role with the mpMRI radiomic features in lesion evaluation. The most common limitations of the studies were the small sample size, retrospective design, lack of validation on external datasets, and unavailability of univocal cut-off values for the selected radiomic features.

## 1. Introduction

Prostate cancer (PCa) is the most frequently diagnosed malignancy in men and the fifth leading cause of death worldwide [1,2]. In primary PCa, risk stratification at staging is crucial to determine prognosis and treatment strategies. The 5-year risk stratification in patients with primary PCa is mainly based on clinical stage, prostate-specific antigen (PSA), and Gleason Score (GS), derived from invasive biopsy samples [3]. Nevertheless, biopsy sampling cannot help in assessing the entire prostate gland and often it might incorrectly grade PCa, especially because of tumor downstaging. Moreover, prostate biopsies are prone to having a false negative rate from 10% up to 30% [4], and transrectal biopsy sampling can be associated with side-effects, such as hematospermia or hematuria [5].

Positron emission tomography (PET) combined with computed tomography (CT) or magnetic resonance imaging (MRI) can help to localize suspicious lesions in the prostate gland by using several prostate-specific radiotracers (i.e., Choline—labelled with either 18F and 11C, 18F-Fluciclovine, and prostate specific membrane antigen-PSMA ligands labelled with 68Ga or 18F), thus providing a valuable tool for the detection of cancer, and for the initial staging of disease [6,7].

Artificial intelligence (AI) is a growing field of computer science that is emerging as a promising adjunct to support physicians in the detection and management of patients with cancer by performing tasks typically requiring human intelligence [8]. In this context, radiomics is a new high-throughput approach to image analysis that aims to quantitatively describe the tumor phenotype through morphological, statistical, and textural characteristics. When applied to medical imaging, such as CT, MRI, or PET scans, radiomics is able to extract quantitative features that might be missed by an expert eye, thereby providing additional and potentially relevant diagnostic information for clinical decision-making in a non-invasive manner [9,10]. The information provided by radiomics might be useful to develop models predictive of cancer diagnosis or prognosis and for risk classification, therefore radiomics is receiving great attention from different medical fields. For instance, in oncology, AI-based models fed with radiomic features, with or without the inclusion of other clinical or histopathological parameters, are built to predict clinical outcomes, such as overall survival, recurrence, risk factors, and others. 

In PCa patients, the potentiality of radiomics has been investigated for the initial staging classification, recurrence detection, and the prediction of metastatic disease using mainly MRI, while data on the utility of PET radiomics are still limited [11]. However, PET radiomics has potential utility in the assessment of tumor heterogeneity [12], although several authors have raised concerns over the robustness and reproducibility of the results [13]. 

The aim of our paper is to perform a systematic review of the literature to provide an overview on the potential application, in terms of the additional value over clinical data, of PET radiomics for the classification of the initial staging of PCa.

## 2. Materials and Methods

**Figure 1:**
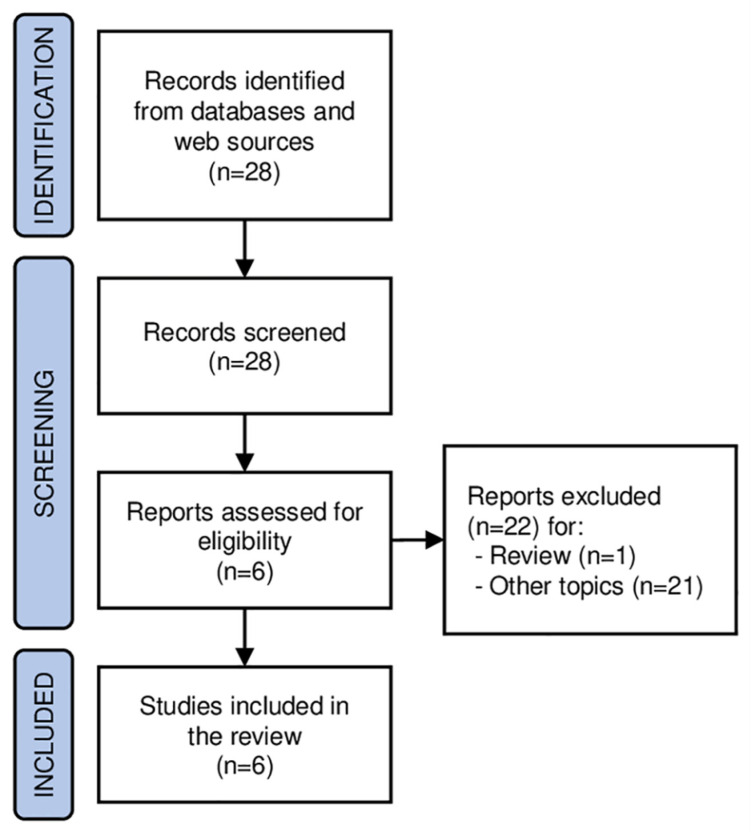
PRISMA statement.

A systematic review was conducted in accordance with the preferred reporting items for systematic reviews guidelines (PRISMA), by P.G., F.M., A.B., and L.E. The authors ran queries to retrieve prospective or retrospective studies on the use of radiomic analysis of PET images at PCa staging in the most relevant databases and web sources (i.e., PubMed, Web of Science, and Scopus). The search query was “prostate AND radiomic* AND PET”. English-language original articles published before July 2021 were considered. After excluding duplicates, papers out of topic, and review articles, the titles and abstracts of the retrieved records were carefully examined. Full texts of the selected articles were obtained, and those written in the English language were analyzed. The references in the articles selected were also screened for additional studies. The following criteria were used to select the studies of interest: (a) PET data were used for radiomic analysis; (b) the PET examination was performed for staging purposes. The flowchart depicting the study selection process is presented in Figure 1 (PRISMA flow-chart). A total of 28 studies were available to be reviewed against the inclusion criteria. Twenty-two studies were excluded based on a review of the title and abstract. Finally, six studies met the inclusion criteria. For each study, the radiomic analysis was assessed based on the radiomic quality score (RQS), a metric that was introduced by Lambin and colleagues in 2017 to specifically evaluate the quality of reporting in the radiomic context [14]. The RQS is in the range of (0–36) (0–100%) and evaluates sixteen different aspects that can be grouped into six main domains: (1) Protocol quality and stability in image and segmentation, (2) feature selection and validation, (3) biologic/clinical validation and utility, (4) model performance index, (5) high level of evidence, and (6) open science and data. For a robust calculation, RQS was blindly computed by two of the authors and discrepancies were discussed to reach a consensus.

## 3. Results

A total of 28 studies were screened for eligibility, of which 6 met the inclusion criteria and were, therefore, selected for further analysis (Figure 1). All studies were based on human patients. The average number of patients included in the studies was 72 (range 52–101). For each study, the list of clinical characteristics is reported in Table 1.

The radiotracers used were [68Ga]Ga-PSMA-11 (in four out of six studies), [18F]DCFPyL (one out of six studies), and [11C]Choline (one out of six studies). Considering the imaging modality, three out of six studies used a PET/CT scanner and the other half a PET/MRI tomograph. 

Zamboglou et al. [15,20] analyzed the role of radiomic features from PSMA PET in a prospective cohort of 20 patients (pts) with intermediate- and high-risk prostate cancer who were selected for a surgical approach. The first study, published in 2019, found that some radiomic features, mainly the “quantized short zones high gray-level emphasis—QSZHGE” (or in IBSI notation “small zones high gray-level emphasis” of the gray level size zone matrix, GLSZM, family, obtained after the application of a quantization algorithm), was able to discriminate low GSs from high ones (i.e., GS < 8 vs. GS >= 8) both considering the GTV derived from histology and the manually segmented one. Moreover, the same variable was also able to discriminate between patients with pathological lymph nodes from those without (i.e., pN1 vs. pN0). The results were internally validated on a retrospective cohort of 40 patients. In the second paper, published in 2021, Zamboglou et al. found that radiomic analysis of PSMA PET data was able to identify missing malignant lesions in the prostate gland, both at the entire and half prostate level. This is important, if we consider that PCa is often multifocal (78% of cases) [16], influencing the correct therapeutic approach, especially in the case of focal treatments. Two PSMA-PET-derived radiomic features, i.e., “[GLSZM] normalized size-zone non-uniformity” and “[GLSZM] small zone emphasis”, derived on PET images after the application of the local binary pattern (LBP), were found to perform excellently in visually unknown PCa detection. The results were validated on an external retrospective cohort of 52 patients. 

Papp et al. [17] evaluated 52 patients with primary PCa who underwent PSMA-PET/MR before any type of therapy. The authors combined radiomic analysis with machine learning to assess the ability of the models to discriminate between low- and high-risk prostate cancer and to predict the biochemical recurrence (BCR) of the disease or the overall patient risk (OPR). They found that a sophisticated machine learning-based model was able to predict the risk with a higher performance than conventional PET metrics (e.g., SUVmax, SUVmean).

The application of radiomic analysis to the hybrid modality PSMA-PET/MR was also investigated by Solari and colleagues [18], specifically for the prediction of the post-surgical GS in primary PCa considering the whole-prostate segmentation performed both on PSMA PET and multiparametric MRI (mpMRI), including T1W, T2W, and ADC map imaging. The 101 pts recruited were grouped according to their GS: Inferior to 8 (60 pts), equal to 8 (23 pts), and superior to 8 (18 pts). Separate models were trained using either radiomic features (107 in total) from a single image type only or features extracted from the combination of a PET image and each MR sequence. The predictions of the best-performing model were compared against biopsy GS (bGS). All radiomic models outperformed two baseline models: One built with patient clinical information and one trained with the volume and maximum-intensity features only. Among single-modality models, the highest performances were achieved by the ADC model (76 ± 6%), even though they were not significantly better than the other models. The combination of PET + ADC radiomic was the best-performing double-modality model (82 ± 5%), significantly better than several other single or double modalities and outperforming bGS. This result demonstrates the synergic role of PET/MRI-based radiomics.

In the study of Tu et al. [19] the authors went beyond the traditional tumor-centric view of radiomic analysis; indeed, they evaluated 77 prostate tumors, but divided the whole prostate organ in three radiomic zones: Zone-1, the metabolic tumor zone; zone-2, the proximal peripheral tumor zone, and zone-3, the extended peripheral tumor zone inside the imaging boundaries of the organ. The radiomic analysis of the three zones was used for risk classification prediction, including the prediction of GS, PSA, TNM, and PFS, and the authors found that these zones have different predicting strengths in classifying risk groups. Zone-1 was superior to the others for PSA-based risk classification prediction, zone-2 and -3 outperformed zone-1 for TNM-based risk, whereas zone-3 was superior to zones 1 and 2 for PFS-based clinical outcome prediction.

In the prospective study by Cysouw and colleagues [21], the data of 76 patients who underwent [18F] DCFPyL PET/CT before radical prostatectomy with extended pelvic lymph node dissection (ePLND) were used to assess if the radiomic-based model applied to primary tumors could predict lymph node involvement (LNI), the presence of any metastases, GS ≥ 8, and the presence of extracapsular extension (ECE). All these high-risk pathological tumor features were predicted by machine-learning-based analysis with significance (*p* < 0.01), thus suggesting that PSMA expression is linked to primary cancer histopathology and metastatic tendency and that radiomic analysis could be integrated into clinical practice to select low-risk patients for whom ePLND would be unnecessary.

**Figure 2:**
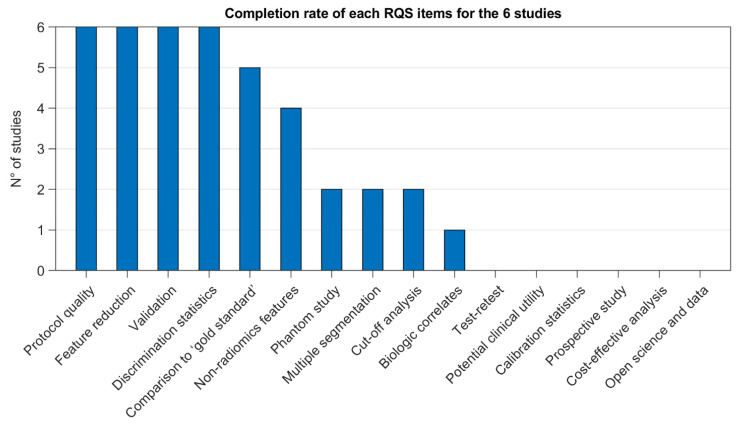
Completion rate of each item of the RQS metric considering all studies. Items evaluate six different domains, i.e., protocol quality, feature selection and validation, clinical validation and utility, model performance, study level of evidence, and open science.

To assess the overall quality of the considered radiomic studies, we adopted the radiomic quality score (RQS) metric. All the considered studies had an RQS lower than 18 (50%) resulting in being non-compliant with the best-practice procedures. In particular, the RQSs ranged from a minimum of 8 (22.22%) for Tu et al. [19] to a maximum of 14 (38.89%) for Zamboglou et al. [15]. The study of Zamboglou et al. [20] received an RQS of 13 (36.11%), followed by Papp et al. [17] and Cysouw et al. [21] with 11 (30.56%) and Solari et al. [18] that scored 10 (27.78%). For all studies, the completion rate of the RQS items is shown in Figure 2. 

Another important aspect to consider in the analysis of these studies is the reproducibility of the results, which requires the possibility of replicating the extraction of radiomic features in detail. The guidelines for radiomic study reporting, which outlines, inter alia, how to report extraction parameters, were published by the Image Biomarker Standardization Initiative (IBSI) [22], an international collaboration formed to standardize the calculation of radiomic features among studies and software. The use of standardized software is of paramount importance to ensure reproducibility. The software that has been used in these studies has different levels of compliance with IBSI in terms of the number of features implemented. For the extraction of radiomic features, four out of six studies used open-source software (i.e., Pyradiomics [23], RaCaT [24], or LIFEx [25]), while two out of six studies used in-house software. All these tools were declared to be IBSI-compliant or participated in the most recent IBSI study. 

On average, 227 features (a range of 50–480) were investigated, including first-, second-, and high-order features, with and without the inclusion of PET-based metabolic parameters such as SUVmax, SUVpeak, SUVmean, and those similar (for five out of six studies).

As far as the methodology is concerned, heterogeneous techniques were adopted. In terms of image processing, two studies employed filtering techniques on the image prior to feature calculation, i.e., wavelet band-pass and the equal-probability histogram quantization algorithm [15] and the local binary pattern [20]. Parameter settings for radiomic feature extraction were rather heterogeneous among studies: Three out of six used isotropic images (two studies resampled the original images to isotropic voxels of 2 × 2 × 2 mm, while in one case, resampling was unnecessary since voxels were already isotropic), five out of six applied intensity discretization with the fixed bin width (FBW) approach (e.g., widths of 0.05 or 0.25 for PET SUV and 5 for ADC) or the fixed bin number (FBN) approach (for MRI data), and five out of six used a 3D feature aggregation method to calculate texture parameters. The study of Tu et al. [19] instead did not report details on feature extraction parameters. 

Regarding tumor delineation, radiomic and clinical features were extracted from the whole prostate in four studies, but other approaches were also proposed. Zamboglou et al. [15] considered manual GTV delineation and the GTVs resulting both from histology and a 40% threshold on SUVmax; Tu et al. [19] defined two more zones based on SUV thresholds (i.e., SUV > 40%, 30% < SUV < 40%), while Cysouw et al. [21] delineated primary tumors using 50–70% background-adapted peak thresholds on images with and without partial-volume correction in order to assess model stability with a threshold.

Concerning modeling and validation techniques, five studies employed machine-learning methods (e.g., random forest, AdaBoost, logistic regression, or support vector machine models) for the classification of GS (*n* = 4 papers), LNI (*n* = 2 papers), low-vs.-high lesion risk (*n* = 1 paper), biochemical recurrence (*n* = 1 paper), overall patient risk (*n* = 1 paper), PSA level (*n* = 1 paper), clinical TNM staging (*n* = 1 paper), progression-free survival (*n* = 1 paper), the presence of ECE (*n* = 1 paper), and metastases (*n* = 1 paper). The study of Zamboglou et al. [20] instead investigated the statistical power of PET-derived radiomic features to discriminate visually undetectable lesions by using the two-tailed Mann–Whitney U test (for non-pairwise testing) or Fisher’s exact test (for the comparison of categorical variables). All works performed feature reduction—to eliminate redundancy among variables—and/or feature selection—to search the optimal feature set and improve classification accuracy. Among the techniques employed, there were analysis of feature correlation, the elimination of features highly correlated with the volume, principal component analysis (PCA), analysis of variance (ANOVA), R-squared feature ranking, wrapper feature selection, or a recursive feature elimination (RFE) algorithm. Two works also analyzed feature robustness to different systems in a separate phantom study prior to including them within the models [15,20]. 

In four studies, models were evaluated in a k-fold cross-validation scheme that divided patients from the same dataset into training and validation cohorts. On the contrary, Zamboglou et al. [15,20] performed a more robust model validation by using two separate cohorts for model development and testing, i.e., they built the model on a prospective cohort of patients and validated the results in a retrospective internal and external cohort, respectively. The most common performance metrics were the area under receiver-operating characteristics curve (AUC) and model accuracy, but the false positive rate, odds-ratio, confidence interval, and other metrics were also adopted. To handle the strong class imbalance, three works resorted the synthetic minority oversampling technique, SMOTE, to generate synthetic samples with interpolated feature values for the less prevalent class [26].

To benchmark the predictive value of PET radiomics against that of basic PET and volume parameters, all studies, except Tu et al. [19], either investigated whether model performance improved with the inclusion of SUV-related features and other clinical parameters among the radiomic features or compared the developed radiomic model against a baseline model trained with clinical parameters only [18,21].

A detailed summary of the radiomic analyses implemented within the selected papers is reported in Table 2.

## 4. Discussion

PCa radiomic analysis is an emerging research field with a high potential to offer non-invasive and longitudinal biomarkers for personalized medicine [27]. Our review is based on a qualitative synthesis of six studies focused on the application of radiomics and AI to PCa staging. Three out of six papers address PET/MRI imaging, a recently introduced modality that has been demonstrated to be superior to PET/CT both in detecting primary PCa lesions and in the assessment of LNI [7]. Interestingly, five out of six articles concerned PSMA-based tracers, probably due to the increased usage of PSMA PET for the staging of primary tumors, which will be further expanded after the recent FDA approval of this imaging modality in some U.S. countries [28].

Measuring intratumoral heterogeneity via images is one of the main targets of radiomic research, which aims to build image-based models for better patient management. The workflow of radiomics follows these steps: (1) Imaging (image acquisition and reconstruction); (2) pre-processing (segmentation and discretization); (3) quantification (feature extraction); and (4) analysis (statistics and/or machine learning methods). The parameters or conditions at each of these steps affect the results [9].

Based on the available data, we can outline some comments. Risk stratification, in terms of GS, can be better determined by the combination of radiomics with PET and MRI data, as clearly reported by Zamboglou [15], Solari [18], and Tu [19]. 

For some years now, mpMRI has been considered an important diagnostic tool for the detection of PCa and it is recommended by the American College of Radiology and European Society of Urogenital Radiology (ESUR) [29,30]. Furthermore, the use of computer-aided diagnosis tools to complement radiologists’ assessments increases sensitivity and specificity in detecting PCa [31]. Combining information from mpMRI and the prostate-specific membrane antigen (PSMA) or 18F- or 11C-Choline PET might offer complementary information in PCa detection, overcoming the limitation of each single technique to identify the entire intraprostatic tumor amount. Nevertheless, even though several papers have already been published on MRI and radiomics in PCa [32], the literature is still scarce regarding radiomics applied to PET examination, thus making it a field that deserves to be further explored.

The opportunity to correctly identify GS can influence therapy, in terms of the duration of androgen deprivation therapy during radiotherapy [5]. Moreover, Zambogou et al. [20] found that machine learning can help in the identification of prostatic lesions otherwise missed by PET with PSMA. From a clinical point of view, we could use these parameters for guiding appropriate focal treatments, thus avoiding under-treatment in patients with multifocal prostate cancer. Zamboglou et al. [15] and Cysouw et al. [21] found that radiomics can also predict the presence of lymph node involvement, therefore guiding tailored treatments. These results can be useful in clinical practice for two main reasons: (1) The identification of the correct GS can be misleading in 20–60% of biopsy specimens, thus affecting the correct management of patients with low/high risk PCa; (2) careful definition of potential lymph node involvement would influence the duration of androgen deprivation therapy during radiotherapy. Currently, nomograms are still used for the definition of the lymphadenectomy extension, but their combination with molecular imaging would be essential in this setting. Prospective trials are mandatory to provide definitive information.

In recent years, many studies have been focused on the role of the tumor microenvironment (TME), which consists of tumor cells, tumor stromal cells, and immune cells, as well as the non-cellular components of the extracellular matrix such as collagen [33]. Understanding the interaction between cancer cells can be used to develop therapeutic strategies to predict and neutralize tactics deployed by cancer cells to survive and resist anti-cancer modalities [34]; in this scenario, the application of radiomic analysis to the tissue surrounding a tumor might provide further insight into the outcome prediction, as demonstrated by Tu [19] in PCa patients, or the response to treatment, as proved in other type of cancers [35,36].

Finally, Papp and Zamboglou [17,20] showed that, in the initial staging of disease, radiomics can predict clinical outcomes such as BCR and OPR. Therefore, the ability of imaging to correctly identify the risk category of each patient has an impact on the long-term outcome.

Despite the promising results of the combination of radiomic and PET data to classify PCa patients at staging, a few comments should be directed to the reproducibility of the methodologies implemented in all studies. Overall, the selected works exhibit limited reproducibility in terms of radiomic feature extraction and only five studies reported details about parameter settings for feature extraction, with an arbitrary level of precision. For example, most of the studies specified the intensity discretization method, but some of them are not clear about the aggregation approach used for textural features (e.g., whether 2D/3D averaged/merged). The results may be dependent on these aspects.

The choice of radiomic software also deserves proper discussion. This aspect is not trivial nor new to the radiomic community, and it is the object of an ongoing international standardization initiative, which was formed to standardize feature formulation across software and studies. We found that two of the studies used private and/or in-house software to calculate IBSI-compliant radiomic features. However, the choice of “available upon request” software further limits the reproducibility of results and is not recommendable.

## 5. Conclusions

Despite the few papers published in the literature so far, preliminary data on the application of radiomic analysis in PCa patients at staging suggest a possible role in pre-operative risk-stratification and the prediction of outcomes together with clinical and histological data. 

Furthermore, the combination of PET and MRI imaging could provide strong support to the hypothesis that radiomics and histology can be linked, and not only in the analysis of the tumor itself, but also that of the tumor microenvironment or even the whole prostate gland, to add useful information in the stratification of patients.

Nevertheless, to confirm the added value of PET radiomics for the initial PCa staging and to accelerate its translation into clinical practice, future works should employ highly standardized software and clearly report detailed indications and comments about their settings, both in terms of feature calculation and image processing, hence making results reproducible. In addition, prospective studies and external validation cohorts would be recommendable to ensure the robustness of the results.

## Figures and Tables

**Table 1:** Clinical characteristics of the selected studies.

Authors, Ref.	Country	n of pts	Median (Range) or Mean (±SD)	Type of RP and Scanner	Risk Category	Results
Zamboglou et al. [14]	Germany	20 (40)	NA	68Ga-PSMA-11 PET/CT	Intermediate and high	QSZHGE can discriminate between high and low GS and pN0 vs. pN1
Zamboglou et al. [15]	Germany	20 (52)	NA	68Ga-PSMA-11 PET/CT	Intermediate and high	Radiomics can detect the presence of multifocal lesions in prostate gland, otherwise missed by visual analysis at PSMA-PET
Papp et al. [16]	Austria	52	64 (59–70)	68Ga-PSMA-11 PET/MR	All	ML and radiomics can predict low vs. high risk, BCR and OS
Solari et al. [17]	Germany	101	68 (63–73)	68Ga-PSMA-11 PET/MR	All	The combination of PET and ADC radiomics is the best performing for GS prediction
Tu et al. [18]	Taiwan	74	69 (52–85)	11C-Choline PET/MR	All	Different radiomic zones in the whole prostate gland have diverse predicting strengths in classifying risk groups
Cysouw et al. [19]	Netherlands	76	66 ± 6	18F-DCFPyL PET/CT	Intermediate and high	Radiomics can predict lymph node involvement and high-risk pathological tumor features

SD = standard deviation, RP = radiopharmaceutical agent, NA = not available; in the brackets are reported the number of patients involved in the validation cohort.

**Table 2:** Radiomic analysis of the selected papers.

Authors, Ref	RQS	Software	N of fts	Params	Delineation	Method
Zamboglou et al. [14]	14 (38.89%)	In-house MATLAB software	133 + 4 SUV-related features	Resampling: None (already isotropic images with 2 × 2 × 2 mm) FBS discretization: 0.05 SUV Aggregation: 3D approach	Manual delineation of GTV from PSMA PET images GTV-Histo resulted from coregistration of histopathology and PET images GTV-40% created as 40% of SUVmax	P cohort: Intraindividual correlations of RFs from different GTVs + feature correlation with GS P&RV cohorts: Uni- and multivariate logistic regression to predict GS and LNI with selected parameters
Zamboglou et al. [15]	13 (36.11%)	PyRadiomics (vers. 2.02)	154 + clinical parameters	Resampling: With and without nearest-neighborhood interpolation to 2 × 2 × 2 mm FBS discretization: 0.05 SUV Aggregation: 3D approach	Manual segmentation of prostate and GTV, based on histology slices coregistered to CT images	Two-tailed Mann–Whitney U test or Fisher’s exact test to evaluate RFs statistical difference between non-PCa-PET areas with or without lesions
Papp et al. [16]	11 (30.56%)	MUW Radiomics Engine (vers. 2.0)	442 + 4 SUV-related features	Resampling: Ordinary Kriging interpolation to 2 × 2 × 2 mm FBS discretization: - PET: 0.05 - T2w: 0.05 - ADC: 5 Aggregation: 3D approach	Use of Hybrid 3D software ver. 4.0.0. and manual correction of segmentations by PET and MRI specialists	Feature reduction with Pearson’s correlation Random forest classifier trained in a 1000-fold Monte Carlo CV scheme R-squared feature ranking
Solari et al. [17]	10 (27.78%)	PyRadiomics	107 + 6 SUV-related features	Resampling: None FBS discretization: - PET: 0.03–1 - ADC: 10–400 FBN discretization: - T1w: 8–256 - T2w: 8–256 Aggregation: 3D approach	Fuzzy-logically adaptive Bayesian (FLAB) segmentation tool of the whole prostate with manual correction	9 SVMs with radial basis function kernel: 4 for single-modality radiomic models, 3 for PET/MRI double-modality, and 2 baseline models needed for comparison RFE method and 6-fold CV scheme
Tu et al. [18]	8 (22.22%)	LIFEx	50	Resampling: NA Discretization: NA Aggregation: NA	Metabolic tumor zone (SUV > 40%) Proximal peripheral tumor zone (30% < SUV < 40%) Whole prostate	Random forest vs. AdaBoost algorithms with 5-fold CV to predict risk classification and clinical outcomes Wrapper feature selection method
Cysouw et al. [19]	11 (30.56%)	RaCaT	480 + 5 clinical parameters tested independently from RFs	Resampling: Trilinear interpolation to 2 × 2 × 2 mm FBS discretization: 0.25 SUV Aggregation: both 2D—3D approaches	Region growing algorithm with background adapted peak threshold varied from 50% to 70% on images with and without PVC	Random forest classifier 3 feature reduction methods (PCA, RFE, ANOVA) to predict LNI and high-risk pathological tumor features

GTV = Gross tumor volume, RFs = radiomic features, FBW = Fixed bin width, FBW = Fixed bin number, PCA = Principal component analysis, RFE = Recursive feature elimination, SVM = Support vector machine, LNI = Lymph node involvement, NA = Not available, P = Prospective cohort, RV = Retrospective validation cohort, PVC = Partial volume correction, CV = Cross validation.
